# Supplementary material for: Hot spot prediction in protein-protein interactions by an ensemble system
Source: BMC Syst Biol. 2018 Dec 31;12(Suppl 9):132. doi: 10.1186/s12918-018-0665-8 (PMC6311905; doi:10.1186/s12918-018-0665-8)
Supplement: Supplementary file 2 — All relevant MATLAB code. A simple Matlab implement of our predictor. (ZIP 19,742 kb) [file 12918_2018_665_MOESM2_ESM.zip › Additional file 2/Read ASA/ASA Property/SKEMPI/2BTFP.txt]

 NetSurfP - Protein Surface Accessibility and Secondary Structure Predictions

  

|  |  |  |
| --- | --- | --- |
|  |  | NetSurfP - Protein Surface Accessibility and Secondary Structure Predictions  Technical University of Denmark |

  


---

```
# For publication of results, please cite:
# A generic method for assignment of reliability scores applied to solvent accessibility predictions.
# Bent Petersen, Thomas Nordahl Petersen, Pernille Andersen, Morten Nielsen and Claus Lundegaard
# BMC Structural Biology 2009, 9:51 doi:10.1186/1472-6807-9-51
#
# Column 1: Class assignment - B for buried or E for Exposed - Threshold: 25% exposure, but not based on RSA
# Column 2: Amino acid
# Column 3: Sequence name
# Column 4: Amino acid number
# Column 5: Relative Surface Accessibility - RSA
# Column 6: Absolute Surface Accessibility
# Column 7: Z-fit score for RSA prediction
# Column 8: Probability for Alpha-Helix
# Column 9: Probability for Beta-strand
# Column 10: Probability for Coil
E A  2BTFP                  1    0.801  88.325  -0.313   0.003   0.003   0.994
E G  2BTFP                  2    0.585  46.039   0.545   0.176   0.004   0.820
B W  2BTFP                  3    0.135  32.516  -1.078   0.694   0.003   0.303
E N  2BTFP                  4    0.407  59.614   1.314   0.782   0.003   0.216
E A  2BTFP                  5    0.512  56.411   0.980   0.879   0.010   0.111
B Y  2BTFP                  6    0.144  30.858   1.389   0.879   0.010   0.111
B I  2BTFP                  7    0.069  12.691   1.181   0.831   0.044   0.125
E D  2BTFP                  8    0.468  67.367   0.116   0.831   0.044   0.125
E N  2BTFP                  9    0.428  62.586  -0.026   0.717   0.014   0.269
B L  2BTFP                 10    0.075  13.733  -0.126   0.522   0.016   0.462
B M  2BTFP                 11    0.094  18.789   0.292   0.354   0.048   0.598
E A  2BTFP                 12    0.329  36.212  -0.813   0.113   0.043   0.844
E D  2BTFP                 13    0.535  77.151  -0.874   0.058   0.017   0.925
E G  2BTFP                 14    0.427  33.589  -1.117   0.018   0.019   0.964
E T  2BTFP                 15    0.382  52.970   0.904   0.056   0.142   0.802
B C  2BTFP                 16    0.039   5.476   0.141   0.022   0.359   0.619
E Q  2BTFP                 17    0.362  64.617   0.763   0.022   0.359   0.619
E D  2BTFP                 18    0.428  61.704   0.970   0.022   0.552   0.426
B A  2BTFP                 19    0.026   2.865   0.338   0.018   0.846   0.136
B A  2BTFP                 20    0.030   3.251   0.627   0.011   0.918   0.071
B I  2BTFP                 21    0.025   4.607   0.711   0.006   0.962   0.032
B V  2BTFP                 22    0.052   8.054   1.038   0.001   0.900   0.099
B G  2BTFP                 23    0.037   2.920  -1.023   0.003   0.718   0.279
B Y  2BTFP                 24    0.200  42.697   0.751   0.004   0.420   0.576
E K  2BTFP                 25    0.590 121.322   0.431   0.004   0.085   0.910
E D  2BTFP                 26    0.589  84.889  -1.466   0.005   0.015   0.979
E S  2BTFP                 27    0.482  56.514  -1.450   0.005   0.015   0.979
E P  2BTFP                 28    0.479  67.928   0.338   0.018   0.047   0.935
B S  2BTFP                 29    0.105  12.283  -0.172   0.022   0.359   0.619
B V  2BTFP                 30    0.051   7.762   0.355   0.021   0.756   0.223
B W  2BTFP                 31    0.056  13.396   0.278   0.018   0.846   0.136
B A  2BTFP                 32    0.026   2.843  -0.367   0.021   0.756   0.223
B A  2BTFP                 33    0.116  12.816  -0.883   0.023   0.655   0.322
B V  2BTFP                 34    0.134  20.611   0.356   0.005   0.262   0.733
E P  2BTFP                 35    0.451  64.068  -1.104   0.005   0.045   0.951
E G  2BTFP                 36    0.571  44.898  -2.043   0.005   0.015   0.979
E K  2BTFP                 37    0.372  76.500  -1.733   0.018   0.019   0.964
E T  2BTFP                 38    0.532  73.816  -0.581   0.018   0.047   0.935
B F  2BTFP                 39    0.278  55.775  -0.839   0.018   0.088   0.893
B V  2BTFP                 40    0.263  40.408  -0.447   0.018   0.088   0.893
E N  2BTFP                 41    0.648  94.896   1.103   0.019   0.141   0.840
B I  2BTFP                 42    0.101  18.759   0.083   0.018   0.088   0.893
E T  2BTFP                 43    0.430  59.669   1.381   0.018   0.047   0.935
E P  2BTFP                 44    0.459  65.075   0.621   0.970   0.001   0.030
E A  2BTFP                 45    0.585  64.489   1.119   0.970   0.001   0.030
B E  2BTFP                 46    0.158  27.672   0.284   0.970   0.001   0.030
B V  2BTFP                 47    0.052   8.008   0.568   0.988   0.000   0.012
E G  2BTFP                 48    0.365  28.757   1.390   0.970   0.001   0.030
E I  2BTFP                 49    0.430  79.642   1.527   0.923   0.002   0.076
B L  2BTFP                 50    0.065  11.865   0.112   0.938   0.007   0.055
B V  2BTFP                 51    0.060   9.283   0.095   0.879   0.010   0.111
E G  2BTFP                 52    0.452  35.588   0.229   0.430   0.016   0.555
E K  2BTFP                 53    0.638 131.216   0.514   0.257   0.016   0.727
E D  2BTFP                 54    0.364  52.524  -0.598   0.053   0.005   0.942
E R  2BTFP                 55    0.459 105.157  -1.035   0.058   0.017   0.925
E S  2BTFP                 56    0.630  73.883   0.169   0.115   0.016   0.868
E S  2BTFP                 57    0.411  48.181  -0.333   0.184   0.043   0.773
B F  2BTFP                 58    0.112  22.498   0.174   0.199   0.152   0.649
B F  2BTFP                 59    0.242  48.589   0.051   0.199   0.152   0.649
B V  2BTFP                 60    0.278  42.713  -1.330   0.118   0.150   0.732
E N  2BTFP                 61    0.455  66.612  -0.984   0.052   0.084   0.864
B G  2BTFP                 62    0.213  16.747  -1.211   0.019   0.141   0.840
B L  2BTFP                 63    0.105  19.207   0.223   0.022   0.552   0.426
B T  2BTFP                 64    0.252  34.883   0.192   0.018   0.846   0.136
B L  2BTFP                 65    0.073  13.330  -0.312   0.001   0.900   0.099
E G  2BTFP                 66    0.320  25.200  -0.511   0.004   0.420   0.576
E G  2BTFP                 67    0.405  31.889  -0.962   0.005   0.262   0.733
E Q  2BTFP                 68    0.355  63.367   0.526   0.002   0.816   0.182
B K  2BTFP                 69    0.218  44.760   0.866   0.001   0.959   0.040
B C  2BTFP                 70    0.045   6.262   0.537   0.001   0.959   0.040
B S  2BTFP                 71    0.057   6.634   0.518   0.001   0.959   0.040
B V  2BTFP                 72    0.032   4.980  -0.100   0.001   0.959   0.040
B I  2BTFP                 73    0.029   5.384   0.603   0.001   0.900   0.099
B R  2BTFP                 74    0.154  35.174   0.441   0.004   0.616   0.381
E D  2BTFP                 75    0.345  49.715  -1.602   0.020   0.205   0.775
E S  2BTFP                 76    0.438  51.380  -1.051   0.056   0.142   0.802
E L  2BTFP                 77    0.310  56.761  -1.506   0.066   0.296   0.638
E L  2BTFP                 78    0.336  61.485  -1.026   0.066   0.296   0.638
E Q  2BTFP                 79    0.483  86.335  -1.615   0.064   0.216   0.721
E D  2BTFP                 80    0.384  55.320  -1.071   0.052   0.084   0.864
E G  2BTFP                 81    0.678  53.351  -1.881   0.018   0.047   0.935
E E  2BTFP                 82    0.561  98.059  -0.068   0.018   0.088   0.893
B F  2BTFP                 83    0.118  23.763   0.093   0.021   0.451   0.528
B T  2BTFP                 84    0.128  17.712  -0.946   0.021   0.756   0.223
B M  2BTFP                 85    0.142  28.354  -0.357   0.018   0.846   0.136
B D  2BTFP                 86    0.257  37.106  -0.734   0.021   0.756   0.223
B L  2BTFP                 87    0.166  30.486  -0.980   0.023   0.655   0.322
B R  2BTFP                 88    0.248  56.884  -0.311   0.022   0.552   0.426
B T  2BTFP                 89    0.241  33.371  -1.825   0.022   0.552   0.426
E K  2BTFP                 90    0.365  75.183   0.023   0.022   0.552   0.426
E S  2BTFP                 91    0.348  40.844  -1.872   0.022   0.359   0.619
E T  2BTFP                 92    0.539  74.718  -1.114   0.019   0.141   0.840
E G  2BTFP                 93    0.623  49.006  -2.356   0.018   0.047   0.935
E G  2BTFP                 94    0.543  42.726  -1.468   0.018   0.019   0.964
E A  2BTFP                 95    0.473  52.180  -1.355   0.018   0.047   0.935
E P  2BTFP                 96    0.511  72.497  -0.913   0.018   0.088   0.893
E T  2BTFP                 97    0.417  57.810  -0.556   0.005   0.262   0.733
B F  2BTFP                 98    0.196  39.277  -0.450   0.005   0.262   0.733
B N  2BTFP                 99    0.058   8.462  -0.415   0.004   0.420   0.576
B I  2BTFP                100    0.019   3.607   1.204   0.011   0.918   0.071
B T  2BTFP                101    0.026   3.648   0.904   0.006   0.962   0.032
B V  2BTFP                102    0.015   2.305   1.549   0.001   0.959   0.040
B T  2BTFP                103    0.060   8.322   0.294   0.006   0.962   0.032
B M  2BTFP                104    0.115  23.112   0.511   0.002   0.816   0.182
B T  2BTFP                105    0.064   8.932  -0.031   0.021   0.279   0.699
E A  2BTFP                106    0.341  37.534   0.808   0.018   0.047   0.935
E K  2BTFP                107    0.224  46.015   0.934   0.020   0.205   0.775
B T  2BTFP                108    0.017   2.400   1.205   0.002   0.816   0.182
B L  2BTFP                109    0.020   3.607   1.325   0.006   0.962   0.032
B V  2BTFP                110    0.026   3.965   0.782   0.001   0.959   0.040
B L  2BTFP                111    0.021   3.863   1.095   0.000   0.983   0.017
B L  2BTFP                112    0.018   3.204   0.666   0.001   0.959   0.040
B M  2BTFP                113    0.061  12.126   0.307   0.001   0.900   0.099
B G  2BTFP                114    0.096   7.563   0.102   0.004   0.514   0.481
E K  2BTFP                115    0.471  96.926   0.018   0.004   0.138   0.858
E E  2BTFP                116    0.547  95.508  -0.781   0.018   0.019   0.964
E G  2BTFP                117    0.504  39.704  -1.727   0.018   0.019   0.964
B V  2BTFP                118    0.139  21.364  -1.231   0.018   0.019   0.964
E H  2BTFP                119    0.312  56.716  -0.668   0.018   0.019   0.964
E G  2BTFP                120    0.298  23.460  -1.231   0.181   0.016   0.803
E G  2BTFP                121    0.319  25.097  -1.597   0.257   0.016   0.727
E M  2BTFP                122    0.313  62.651  -0.424   0.339   0.016   0.645
B I  2BTFP                123    0.028   5.180  -0.063   0.622   0.015   0.363
B N  2BTFP                124    0.145  21.169   0.514   0.858   0.002   0.139
E K  2BTFP                125    0.454  93.450   1.226   0.970   0.001   0.030
B K  2BTFP                126    0.123  25.198   1.264   0.970   0.001   0.030
B C  2BTFP                127    0.013   1.797   1.527   0.988   0.000   0.012
E Y  2BTFP                128    0.259  55.284   0.498   0.988   0.000   0.012
E E  2BTFP                129    0.416  72.745   1.870   0.988   0.000   0.012
B M  2BTFP                130    0.036   7.164   0.775   0.970   0.001   0.030
B A  2BTFP                131    0.039   4.342  -0.261   0.970   0.001   0.030
E S  2BTFP                132    0.506  59.338   1.772   0.970   0.001   0.030
B H  2BTFP                133    0.208  37.835   1.067   0.970   0.001   0.030
B L  2BTFP                134    0.038   7.031  -0.676   0.970   0.001   0.030
E R  2BTFP                135    0.384  87.867   1.225   0.923   0.002   0.076
E R  2BTFP                136    0.729 166.987   0.645   0.858   0.002   0.139
E S  2BTFP                137    0.392  45.942   0.559   0.406   0.004   0.590
E Q  2BTFP                138    0.685 122.341  -1.951   0.053   0.005   0.942
E Y  2BTFP                139    0.554 118.368  -0.919   0.003   0.003   0.994

Explain the output.  Go back.
```

---
